# Supplementary material for: Venomix: a simple bioinformatic pipeline for identifying and characterizing toxin gene candidates from transcriptomic data
Source: PeerJ. 2018 Jul 31;6:e5361. doi: 10.7717/peerj.5361 (PMC6074769; doi:10.7717/peerj.5361)
Supplement: Supplemental Information 3 [file peerj-06-5361-s003.gz › FinalOutput_E-6/Kunitz-type_serine_protease_inhibitor_2_4/finaltree.pdf]

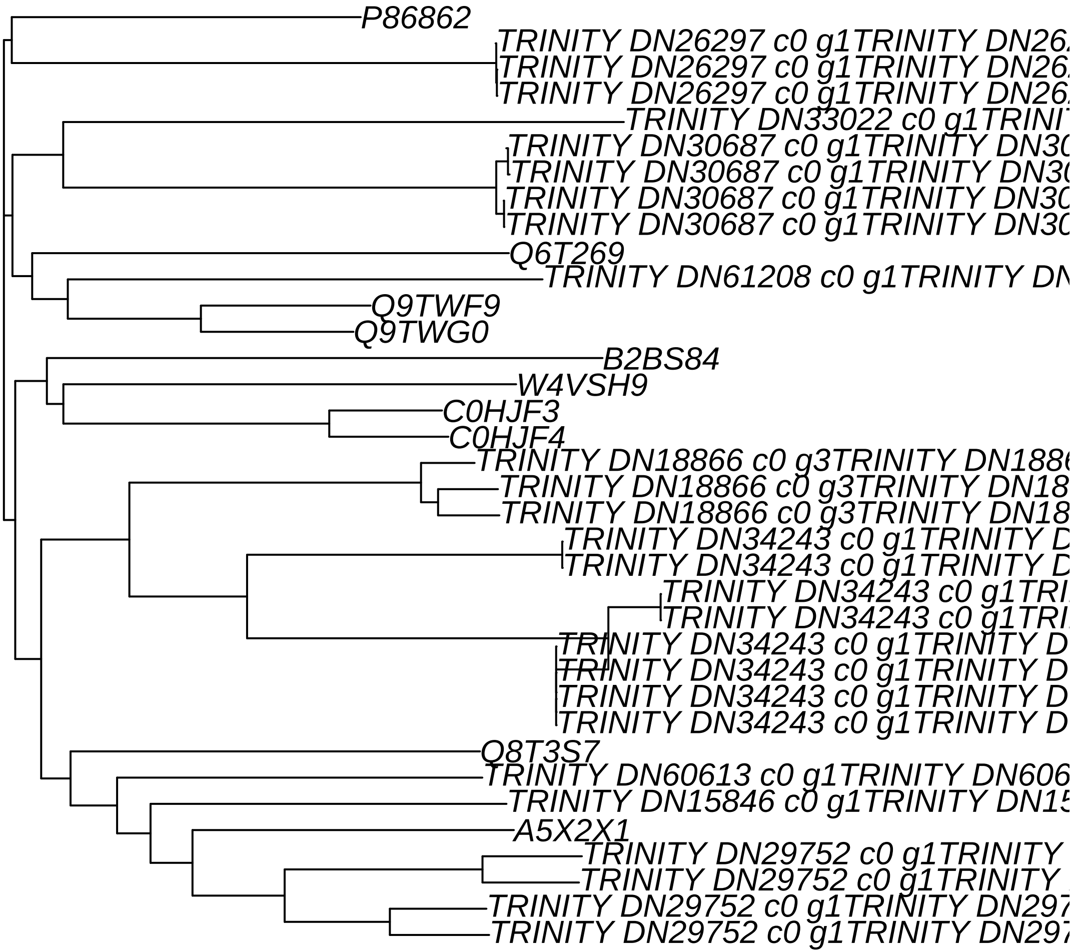
P86862
TRINITY DN26297 c0 g1TRINITY DN26
TRINITY DN26297 c0 g1TRINITY DN26
TRINITY DN26297 c0 g1TRINITY DN26
TRINITY DN33022 c0 g1TRINIT
TRINITY DN30687 c0 g1TRINITY DN30
TRINITY DN30687 c0 g1TRINITY DN30
TRINITY DN30687 c0 g1TRINITY DN30
TRINITY DN30687 c0 g1TRINITY DN30
Q6T269
TRINITY DN61208 c0 g1TRINITY DN
Q9TWF9
Q9TWG0
B2BS84
W4VSH9
C0HJF3
C0HJF4
TRINITY DN18866 c0 g3TRINITY DN188
TRINITY DN18866 c0 g3TRINITY DN18
TRINITY DN18866 c0 g3TRINITY DN18
TRINITY DN34243 c0 g1TRINITY D
TRINITY DN34243 c0 g1TRINITY D
TRINITY DN34243 c0 g1TRI
TRINITY DN34243 c0 g1TRI
TRINITY DN34243 c0 g1TRINITY D
TRINITY DN34243 c0 g1TRINITY D
TRINITY DN34243 c0 g1TRINITY D
TRINITY DN34243 c0 g1TRINITY D
Q8T3S7
TRINITY DN60613 c0 g1TRINITY DN606
TRINITY DN15846 c0 g1TRINITY DN15
A5X2X1
TRINITY DN29752 c0 g1TRINITY
TRINITY DN29752 c0 g1TRINITY
TRINITY DN29752 c0 g1TRINITY DN297
TRINITY DN29752 c0 g1TRINITY DN297
